# TNF-α-mediated downregulation of CD36 and phagocytic impairment of alveolar macrophages via upregulation of ADAM17 in asthma

**DOI:** 10.3389/fimmu.2025.1663513

**Published:** 2025-10-16

**Authors:** Xingyue Liu, Ya Li, Feifei Shang, Minzhu Niu, Jiaqi Yan, Minyu Xie, Xiangnan Tao, Han Huang, Wenwen Wu, Shu Dong, Yingzi Chen, Fan Wu, Shujun Guo, Yulin Du, Mengqing Hua, Yanmei Hao, Chuanwang Song

**Affiliations:** ^1^ Anhui Province Key Laboratory of Immunology in Chronic Diseases, Bengbu Medical University, Anhui, China; ^2^ School of Clinical Medicine, Bengbu Medical University, Anhui, China; ^3^ Clinical Laboratory, The Second Affiliated Hospital of Bengbu Medical University, Anhui, China; ^4^ School of Laboratory Medicine, Bengbu Medical University, Anhui, China

**Keywords:** asthma, alveolar macrophages, phagocytosis, CD36, ADAM17, TNF-α

## Abstract

**Background:**

Alveolar macrophages (AMs) are specialized phagocytes in the airways that play a crucial role in maintaining bronchoalveolar homeostasis through phagocytosis, the clearance of apoptotic cells. However, the characteristics and molecular mechanisms of AMs-mediated phagocytosis during the pathogenesis of asthma remain poorly characterized.

**Methods:**

An ovalbumin (OVA)-induced asthma model was established in mice through intraperitoneal sensitization followed by intranasal challenge. AMs were isolated from the bronchoalveolar lavage fluid of control and OVA-induced mice using adherence-based purification. The phagocytic capacity of AMs, as well as the expression levels of CD36 and ADAM17, were quantified by flow cytometry.

**Results:**

A significant reduction in both phagocytic efficiency and CD36 expression was found in the AMs of OVA-induced mice compared to control mice. Blockade of CD36 resulted in a marked decline in the phagocytic efficiency of normal AMs. Expression of ADAM17 was found to be notably elevated on the surface of AMs from OVA-induced mice compared to controls. Knockdown of ADAM17 led to a substantial increase in CD36 expression and a corresponding increase in phagocytic efficiency. Stimulation with tumor necrosis factor-α (TNF-α) resulted in a significant upregulation in ADAM17 and marked downregulation in CD36 expression levels, as well as impaired the phagocytic efficiency of AMs. Importantly, ADAM17 knockdown attenuated the TNF-α-mediated downregulation of CD36 expression and the associated impairment of phagocytic capacity in AMs.

**Conclusion:**

AMs from OVA-induced mice displayed significantly impaired phagocytic capacity. Airway TNF-α upregulated ADAM17, which in turn downregulated CD36 expression on AMs, ultimately suppressing their phagocytic function.

## 1 Introduction

Asthma, a chronic inflammatory disease of the airways, affects approximately 300 million people worldwide, and its prevalence continues to rise (1). The clinical manifestations of asthma, including coughing, wheezing, shortness of breath, and chest tightness, are closely associated with airway inflammation, hyperresponsiveness, and remodeling (2). Asthma is classified into two endotypes: type 2-high and type 2-low (3). In type 2-high asthma, chronic airway inflammation is driven by Th2 cells and type 2 innate lymphoid cells, mediated by type 2 cytokines (interleukin-4 (IL-4), IL-5, and IL-13). This leads to dysregulated proliferation and activation of eosinophils, macrophages, and dendritic cells, as well as enhanced epithelial injury and apoptosis (4). The accumulation of apoptotic cells in the airways and their impaired clearance further exacerbates the inflammatory response (4).

Phagocytosis, which involves cellular uptake of particles larger than 0.5 μm in diameter, is a critical mechanism of innate immunity and enables the immune system to eliminate pathogens and apoptotic cells, thereby preventing infections and inflammation (5). phagocytosis is mediated by both professional and non-professional phagocytes, with macrophages, neutrophils, and dendritic cells being the primary professional phagocytes (6). Alveolar macrophages (AMs) are specialized phagocytes in the bronchoalveolar region and constitute the first line of defense against pulmonary infections. They eliminate pathogens through the phagocytosis of invading bacteria and viruses and prevent dissemination of infections. In addition, AMs clear apoptotic cells, attenuate inflammatory cascades, promote tissue repair, and maintain bronchoalveolar homeostasis (7) (8).

The phagocytic capacity of AMs is dynamically regulated by various diseases and environmental factors (9). In a lipopolysaccharide-induced lung injury model, for example, reduced ADAM17 expression significantly potentiates the phagocytosis of apoptotic neutrophils by AMs (10), while CD36-deficient AMs are found to display marked reduced phagocytic clearance of the pathogen during acute intrapulmonary infection with *Klebsiella pneumoniae* (11). Emerging evidence has indicated that AMs exhibit a significantly impaired phagocytic capacity in chronic obstructive pulmonary disease (12). Finally, acute ozone-induced pulmonary inflammation has been shown to impair the phagocytosis of apoptotic neutrophils by AMs through MERTK receptor deficiency (13). However, the characteristics and underlying mechanisms of phagocytosis by AMs during the pathogenesis of asthma remain poorly characterized.

In this study, we constructed a type 2-high asthma model in mice via ovalbumin (OVA) sensitization and challenge and examined changes in the phagocytic capacity of AMs in OVA-induced mice, as well as investigated the underlying mechanisms.

## 2 Materials and methods

### 2.1 Animals

Specific pathogen-free female BALB/c mice were obtained from Jiangsu Qinglongshan Biotechnology Co., Ltd. All animals were group-housed under controlled conditions with a 12-hour light/dark cycle, maintained at 22 ± 2 °C with 40−60% relative humidity. All experimental procedures were approved by the Institutional Animal Care and Ethics Committee of Bengbu Medical University.

### 2.2 Construction of an asthma model

Nine-week-old female mice were randomly assigned to the following groups: control (PBS-treated), OVA, TNF-α antibody blockade (TNF-Ab), and isotype control (OVA + Iso Ab). Mice in the OVA group received intraperitoneal injections of 100 μg OVA (Sigma-Aldrich, USA) and 2 mg Al(OH)_3_ adjuvant (Thermo Fisher Scientific, USA) dissolved in 200 μL PBS on days 0, 3, and 5. The control group received an equal volume of PBS only. On days 10, 12, and 14, airway challenge was performed via intranasal instillation of 50 μg OVA in the OVA group. The control group received an equal volume of PBS only. In the TNF-Ab group, a TNF-α neutralizing antibody (26405-1-AP, Proteintech, China) was administered intranasally at a dose of 300 μg/kg 12 hours before each airway challenge. The isotype control group (OVA + Iso Ab) received an equivalent amount of isotype control antibody following the same protocol. All assessments were conducted 24 hours after the final challenge. Endpoints were assessed 24 hours post-final challenge.

### 2.3 Paraffin embedding and hematoxylin-eosin staining

Lung tissues were fixed in 4% paraformaldehyde (PFA), dehydrated through an ethanol gradient, cleared in xylene, and embedded in paraffin. Sections (5 μm) were floated on a 45 °C water bath and dried. For H&E staining, sections were deparaffinized, rehydrated, stained with hematoxylin (3 min) and eosin Y (2 min), differentiated in acid ethanol, stained blue in ammonia water, dehydrated, and mounted. Samples were examined under light microscopy.

### 2.4 Detection of airway resistance

Airway resistance (RL) was measured using an invasive pulmonary function system. Mice were anesthetized with amobarbital sodium (40 mg/kg), intubated, and ventilated (150 breaths/min, 10 ml/kg tidal volume). Aerosolized acetylcholine chloride (0–30 mg/mL) (MCE, USA) was administered, and airway hyperresponsiveness was assessed by measuring changes in the respiratory system resistance (Rrs).

### 2.5 Lung wet/dry weight ratio

The left lung was rinsed, blotted, and weighed (W), dried at 65 °C for 72 hours, then reweighed (D). The wet/dry weight ratio (W/D) was calculated as W/D.

### 2.6 Enzyme-linked immunosorbent assay

The concentrations of IL-4 (Cusabio, China), IL-5 (Cusabio, China), IL-1β (Cusabio, China), and TNF-α (Invitrogen, USA) were quantified using commercial ELISA kits according to the manufacturer’s protocols. Optical density (OD) was measured at 450 nm using a microplate reader.

### 2.7 Isolation of bronchoalveolar lavage fluid

Mice were humanely killed, and bronchoalveolar lavage was performed using ice-cold PBS. BALF was centrifuged, red blood cells were lysed, and cells were resuspended in RPMI-1640 with 10% FBS. AMs were purified by adherence at 37 °C for 2 hours, and Siglec-F^+^ (Invitrogen, USA)/CD11c^+^ (Invitrogen, USA) cell purity (>95%) was confirmed by flow cytometry (**Supplementary Figure S1**).

### 2.8 Measurement of soluble CD36

sCD36 in BALF: BALF was centrifuged at 300 × g for 10 min to remove cells and debris. The resulting supernatant was collected and subjected to ELISA (Cusabio, China) for detection.

sCD36 in cell culture supernatant: AMs isolated from BALF were seeded in 6-well plates. After 48 h of siRNA transfection, the culture medium was collected, centrifuged at 300 × g for 5 min to remove cellular components, and analyzed by ELISA.

### 2.9 Eosinophil count

BALF was obtained from mice. After centrifugation, the cell precipitates were resuspended in PBS. Wright’s staining was used to produce and stain cell smears. Lastly, a 400× magnification microscope was used to count the eosinophils.

### 2.10 Induction of apoptotic cells

After sufficient AEC or Jurkat cells were harvested, they were centrifuged and the supernatant was discarded. The cell pellet was resuspended in PBS, and camptothecin (Beyotime, China) was added to a final concentration of 10 µM. The cells were then incubated at 37 °C for 24 h. After induction, the cells were washed with PBS and resuspended again. Pre-warmed Cell-Tracker Green CMFDA (Maokang Biotech, China) was added to 5 µM, and the suspension was incubated at 37 °C in a CO_2_ incubator in the dark for 24 h. Finally, the cells were washed with PBS to obtain the apoptotic population (**Supplementary Figure S2**).

### 2.11 Phagocytosis assay

The phagocytosis of apoptotic cells by AMs was examined by plating AMs (isolated from the BALF) into 24-well plates at a concentration of 1×10^5^ cells per well. After adherence, cells were incubated with Cell-Tracker Deep Red (Maokang Biotech, China) live cell marker at 37 °C in an atmosphere of 5% CO_2_ for 40 minutes. Subsequently, 3×10^5^ apoptotic AEC/Jurkat cells labeled with Cell-Tracker Green CMFDA (Maokang Biotech, China) were added to each well and co-cultured for 6 hours. Cells were then blown into flow cytometry tubes, washed twice with PBS containing 1% FBS, centrifuged at 100 × g for 5 minutes, and fixed with 300 μL 4% PFA. The phagocytosis of apoptotic cells by AMs was then analyzed using a Cytek DxP Athena flow cytometer. Gating strategy: AMs were first gated based on FSC/SSC characteristics, followed by exclusion of doublets using FSC versus FSCW. Then, AMs were identified using a Cell-Tracker Deep Red single-stained sample to set the gate in the APC channel. The phagocytic capacity of this gated population was finally assessed in the FITC channel by measuring the percentage of cells that had ingested CMFDA-labeled apoptotic cells. Corresponding representative gating plots are provided in **Supplementary Figure S3**.

### 2.12 Flow cytometry analysis of surface molecule expression on AMs

To evaluate the expression of CD36, MERTK, and ADAM17 on the surface of AMs, cells were incubated with anti-CD36 (Invitrogen, USA), anti-MERTK (Invitrogen, USA), and anti-ADAM17 (Bioss, China) primary antibodies, and isotype-matched control antibodies on ice for 30 minutes. After staining, cells were fixed with 1% PFA and analyzed by flow cytometry. Data are expressed as mean fluorescence intensity (MFI).

### 2.13 RNA extraction and qRT-PCR assays

Total RNA from AMs was extracted and converted into cDNA using a reverse transcription kit. qRT-PCR was conducted with Roche’s LightCycler 480 and a quantitative kit, following the manufacturer’s instructions. All the primers used are shown in **Supplementary Table S1**.

### 2.14 The siRNA transfection

A total of 5×10^5^ AMs were seeded in 6-well plates and cultured until they reached approximately 50% confluence. Adam17-targeted small interfering RNA (siRNA) or non-targeting control siRNA (Sangon Biotech, China) was transfected into the cells using Lipofectamine 2000 transfection reagent (Invitrogen, USA), following the manufacturer’s instructions. The medium containing low serum was replaced 6 hours post-transfection, and subsequent experiments were performed after an additional 42 hours of incubation. All the target sequences were shown in **Supplementary Table S2**.

### 2.15 Western blotting

Membrane proteins were extracted using a commercial membrane protein extraction kit (Proteintech, China), determining protein concentration via a BCA assay, and separating equal protein amounts on SDS-PAGE gels before transferring them to PVDF membranes. The membranes were blocked with 5% skim milk for 2 hours at room temperature, then incubated overnight with primary antibodies at 4 °C, followed by a 1-hour incubation with HRP-labeled secondary antibodies. Protein bands were detected using the Gel Doc XR system and analyzed with Image Lab software. The antibodies used in this study are as follows: anti-ADAM17 (# bs-4236R, Bioss), Na^+^/K^+^-ATPase (#CY5159, Abways).

### 2.16 Statistical analysis

Data are presented as the mean ± SD and were analyzed using GraphPad Prism 9. For comparisons involving multiple groups, one-way (or two-way) analysis of variance (ANOVA) followed by Tukey’s *post hoc* test was used. Comparisons between two groups were performed using Student’s *t*-test. A P-value < 0.05 was considered to be statistically significant.

## 3 Results

### 3.1 The phagocytic capacity of AMs is markedly reduced in OVA-induced mice

**Figure 1:**
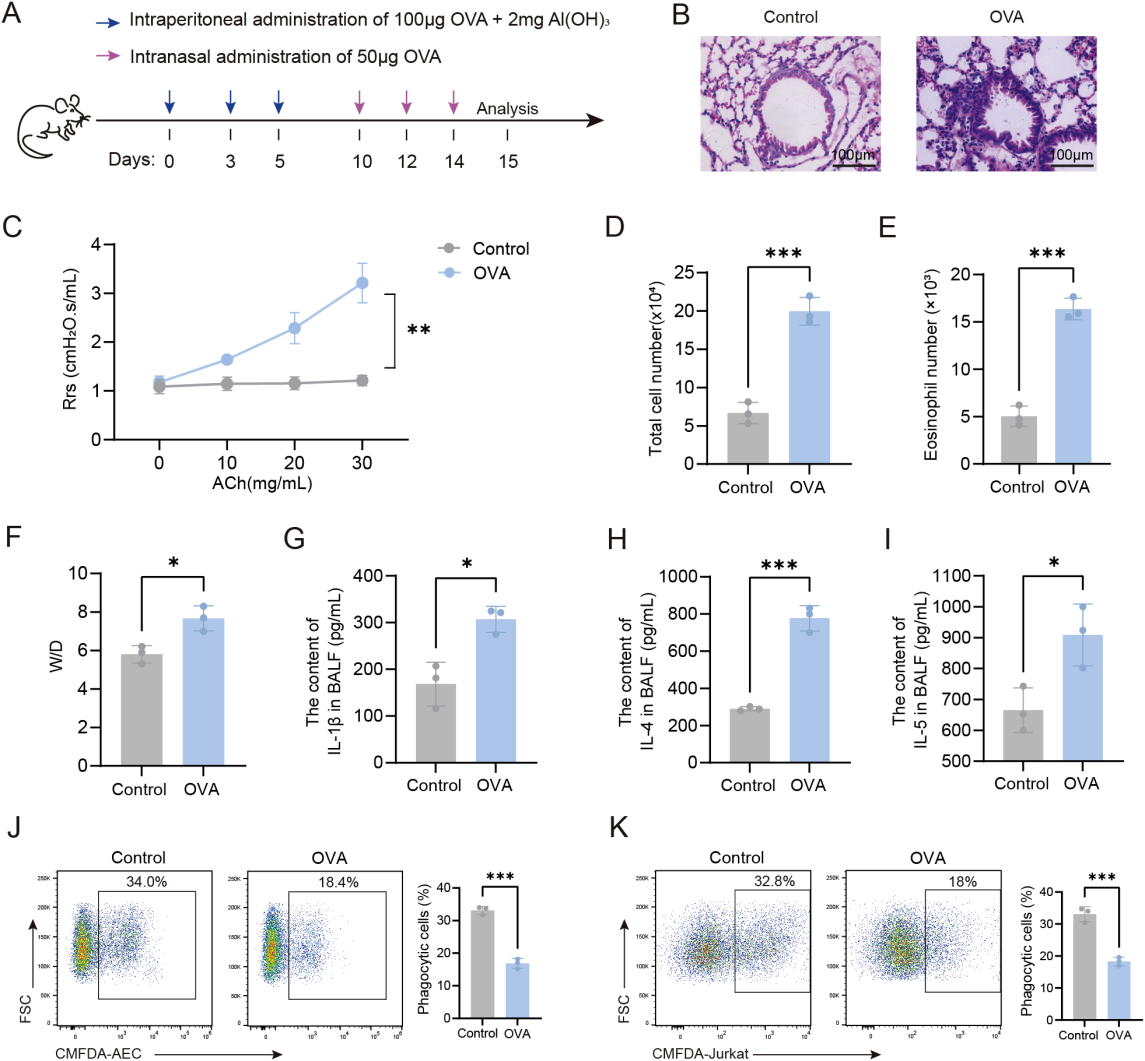
Impaired phagocytic capacity of AMs in OVA-induced mice. **(A)** Experimental Timeline for the Induction of an OVA-Induced Asthma Model in Mice. **(B)** HE-stained sections of mouse lung tissue (×200). **(C)** Airway resistance in mice was measured using invasive techniques. **(D)** Total cell count in BALF of mice was quantified. **(E)** Eosinophil count in BALF from normal control and OVA-induced mice was determined. **(F)** The wet-to-dry weight ratio of the left lung in mice was measured. **(G-I)** Levels of IL-1β, IL-4, and IL-5 in BALF were detected by ELISA. **(J)** Phagocytosis of apoptotic AEC cells by AMs from each group of mice was assessed by flow cytometry. **(K)** Phagocytosis of apoptotic Jurkat cells by AMs was assessed by flow cytometry. For **(J, K)** gating strategy is described in Methods. In **(J, K)** the left panels display representative flow cytometry plots, and the right panels show statistical graphs. Control: Normal control mice, OVA: OVA-induced mice. n=3, ^*^ *P* < 0.05, ^**^ *P* < 0.01, ^***^ *P* < 0.001.

We developed an OVA-induced asthmatic mouse model to examine the phagocytic function of AMs in asthma (**Figure 1A**). we found that, compared to control mice, OVA-induced mice displayed significant airway inflammation, increased airway resistance, and increased total cell counts, eosinophil numbers, and pro-inflammatory cytokine levels (IL-1β, IL-4, and IL-5) in the BALF (**Figures 1B–I**), indicating that the murine OVA-induced model used in this study represented the type 2-high endotype. Our flow cytometry data revealed that AMs from OVA-induced mice displayed significantly impaired phagocytosis of both apoptotic AEC and apoptotic thymocytes (Jurkat cells) compared to control AMs (**Figures 1J, K**). Together, these results indicated that the phagocytic capacity of AMs was markedly reduced in OVA-induced mice.

### 3.2 The impaired phagocytic capacity of AMs from OVA-induced mice is associated with the downregulation of CD36 expression levels

**Figure 2:**
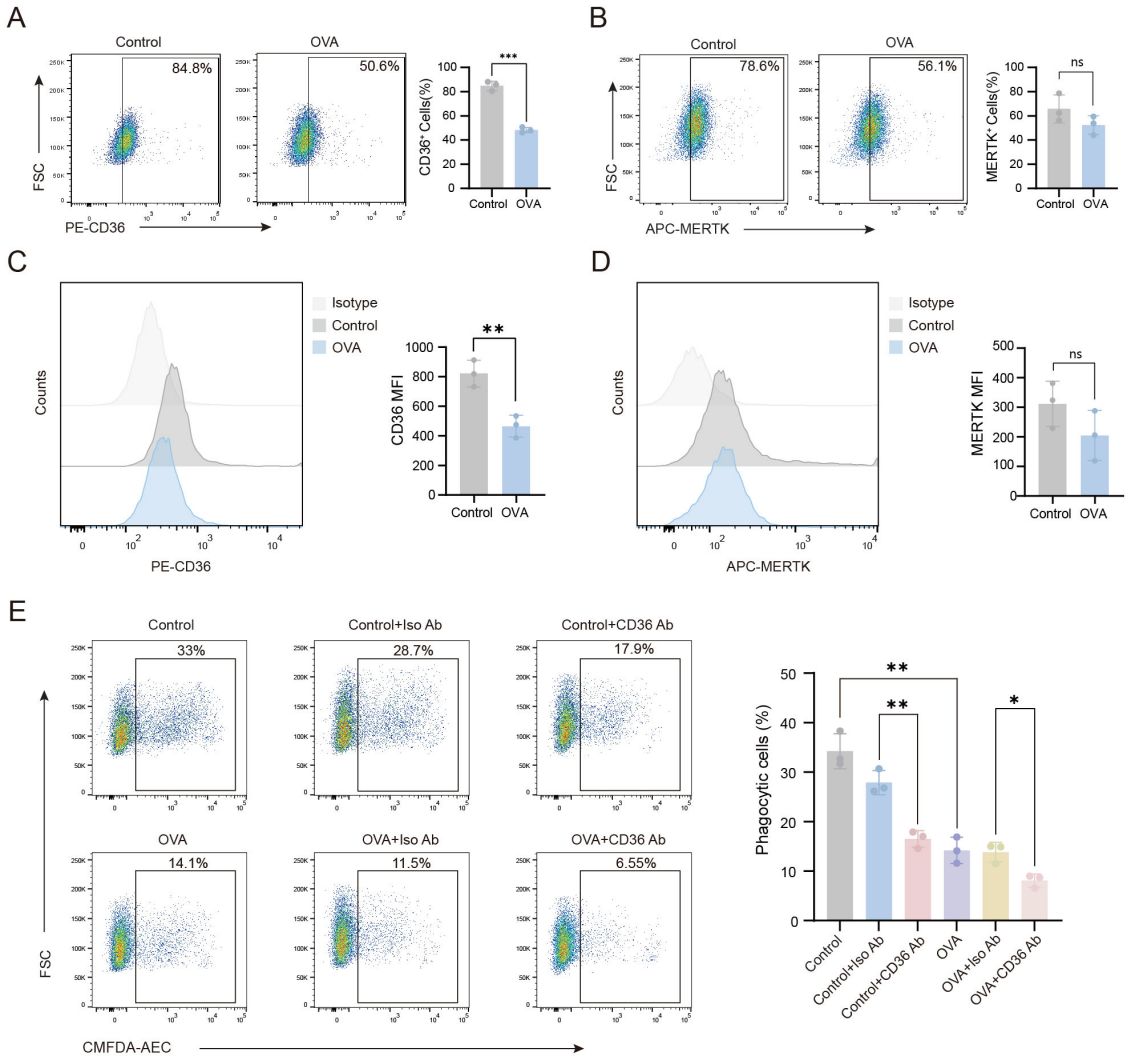
The impaired phagocytic capacity of AMs in OVA-induced mice is associated with the downregulation of CD36 receptor expression levels. **(A–D)** Flow cytometry was performed to analyze the expression of CD36 and MERTK on the surface of AMs from mice in the various treatment groups. **(E)** AMs from each group were treated with CD36 blocking antibody, then the phagocytosis of apoptotic cells by AMs was assessed. **(A–D)** Gating strategy: CD36^+^ and MERTK^+^ cells were gated based on unstained negative controls. For **(E)** gating strategy is described in Methods. Representative flow cytometry plots are shown in the left panels, and statistical graphs are shown on the right. Control: normal control group, OVA: OVA-induced group, Control+Iso Ab: normal control group treated with isotype antibody, Control+CD36 Ab: normal control group treated with CD36 antibody, OVA+Iso Ab: OVA-induced group with isotype antibody, OVA+CD36 Ab: OVA-induced group treated with CD36 antibody. n=3, ^*^ *P* < 0.05, ^**^ *P* < 0.01, ^***^ *P* < 0.001.

Macrophage phagocytosis is mediated by receptors that directly or indirectly bind phosphatidylserine (PS). Thus, we assessed the expression of CD36, a direct PS-binding receptor, in AMs. Our flow cytometry data showed that CD36 surface expression levels were significantly downregulated on the AMs of OVA-induced mice compared to control mice (**Figures 2A–D**). In contrast, no significant difference in the expression of MERTK, an indirect PS-binding receptor, was observed between AMs isolated from control and OVA-induced mice. Next, we investigated the functional role of CD36 in phagocytosis by treating AMs with a CD36-blocking antibody. Blockade of CD36 led to significantly impaired phagocytosis by AMs from both control and OVA-induced mice (**Figure 2E**), confirming the essential role of CD36 in phagocytosis. Together, these findings suggested that the impaired phagocytic capacity of AMs in OVA-induced mice may be primarily attributed to reduced CD36 expression.

### 3.3 ADAM17 negatively regulates the phagocytic capacity of AMs through downregulation of CD36 expression on the surface of AMs

**Figure 3:**
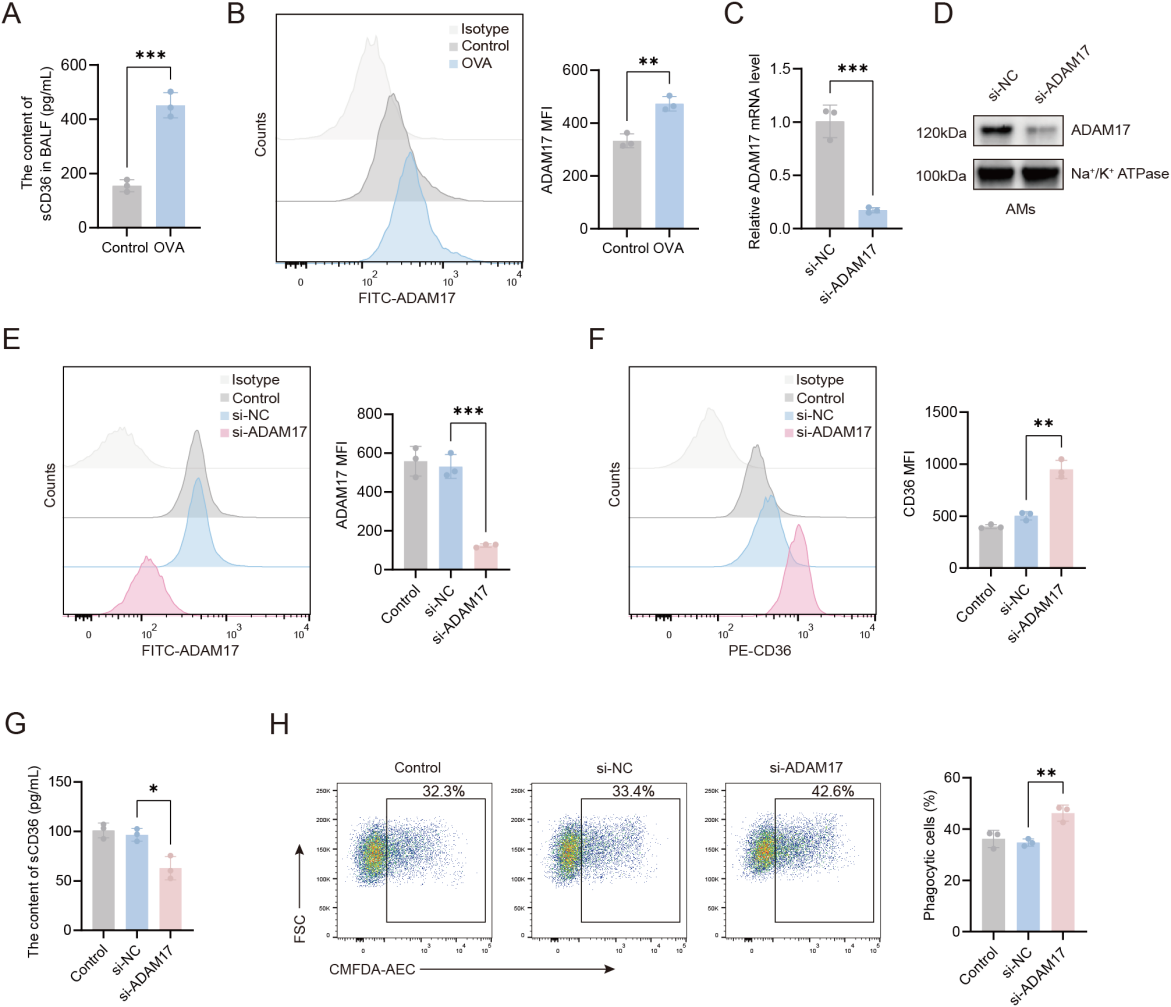
ADAM17 suppresses AMs-mediated phagocytosis through the downregulation of CD36 surface expression. **(A)** Levels of sCD36 in BALF were detected by ELISA. **(B)** Flow cytometry was used to assess ADAM17 expression levels on AMs from control and OVA-induced mice. **(C)** qRT-PCR was used to detect the expression level of the ADAM17 gene on AMs from control mice 48 hours after transfection with ADAM17-siRNA. **(D)** Western blot was used to detect ADAM17 protein expression levels in the cell membrane of AMs from control mice 48 hours after transfection with ADAM17-siRNA. **(E)** Flow cytometry was used to assess ADAM17 expression levels on AMs from control mice 48 hours after transfection with ADAM17-siRNA. **(F)** Flow cytometry was used to examine the effects of ADAM17 knockdown on CD36 expression levels on AMs from control mice 48 hours after transfection with ADAM17 siRNA. **(G)** ELISA was used to measure the levels of sCD36 in the cell supernatant of AMs from control mice 48 hours after transfection with ADAM17-siRNA. **(H)** Flow cytometry was used to assess the effects of ADAM17 knockdown on AMs-mediated phagocytosis in AMs from control mice 48 hours after transfection with ADAM17-siRNA. **(B, E, F)** Gating strategy: The gating strategy for CD36 and ADAM17 was the same as that described for **Figures 2A–D**. For **(H)** gating strategy is described in Methods. **(B, E, F, H)** the left panels show representative flow cytometry plots, and the right panels show statistical analyses. Control: control group; si-NC: siRNA negative control (scrambled sequence) group; si-ADAM17: ADAM17-siRNA group. n=3, *p < 0.05, **p < 0.01, ***p < 0.001.

To investigate the reduced CD36 expression on AMs in OVA-induced mice, we measured soluble CD36 (sCD36) in BALF. The sCD36 levels were significantly higher in the OVA group than in controls (**Figure 3A**), suggesting CD36 cleavage and release into body fluids. ADAM17, a membrane-bound metalloprotease, modulates the expression of phagocytic receptors on the cell surface, thereby regulating cellular phagocytic capacity (14). Given ADAM17’s enzymatic activity in cleaving cell surface receptors, we assessed its expression on the surface of AMs. Our flow cytometry data revealed that there was a marked upregulation in the expression of ADAM17 on the surface of AMs from OVA-induced mice compared to control mice (**Figure 3B**).

To investigate the regulatory role of ADAM17 in AMs, we knocked down ADAM17 in AMs using small interfering RNA, and the knockdown efficiency is shown in **Figures 3C–E**. The expression of ADAM17 in AMs was reduced by approximately 70% following transfection with the specific siRNA. To further clarify the effect of ADAM17 on phagocytosis-related molecules, we examined the expression of CD36 on the surface of AMs. As shown in **Figure 3F**, ADAM17 knockdown significantly increased surface CD36 expression on AMs. Furthermore, the concentration of sCD36 in the culture supernatant of this group was markedly reduced compared to the control group (**Figure 3G**). The elevated sCD36 levels in BALF (**Figure 3A**) reflected the total CD36 shedding in the airway microenvironment *in vivo*. These experiments demonstrate that ADAM17 can mediate the proteolytic cleavage of CD36. The effects of ADAM17 on phagocytosis were next examined in AMs transfected with ADAM17-siRNA. We found that ADAM17 knockdown markedly enhanced the phagocytic capacity of AMs compared to scrambled siRNA controls (**Figure 3H**), suggesting that ADAM17 acted as a negative regulator of phagocytosis. These results indicated that ADAM17 inhibited phagocytosis by AMs through downregulation of CD36 surface expression.

### 3.4 TNF-α-mediated downregulation of CD36 on AMs via ADAM17 suppresses the phagocytic capacity of AMs.

**Figure 4:**
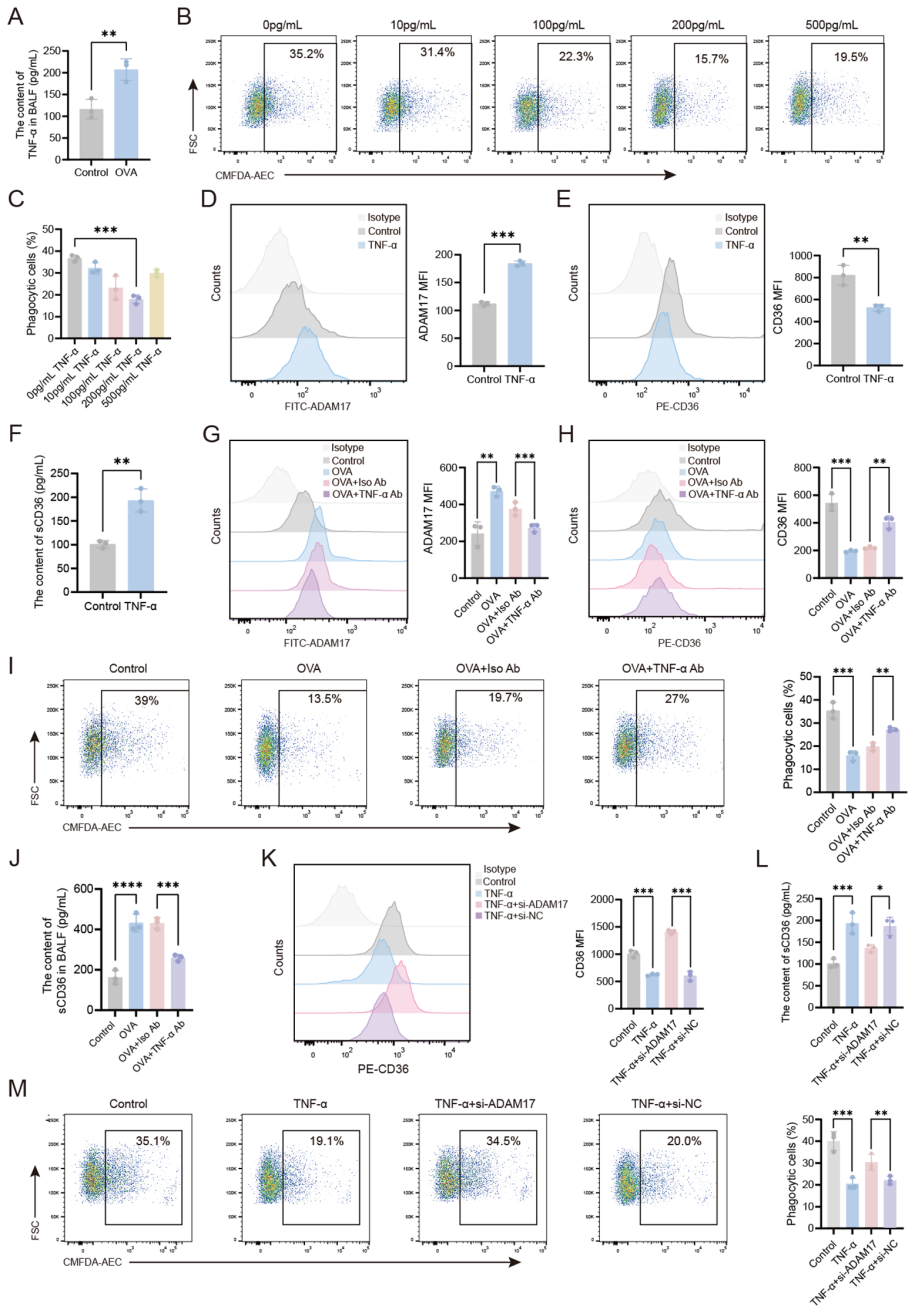
TNF-α-mediated downregulation of CD36 on AMs via ADAM17 impairs the phagocytic function of AMs. **(A)** TNF-α levels in the BALF of healthy control and OVA-induced mice were measured by ELISA. **(B, C)** AMs from normal mice were stimulated with varying concentrations of TNF-α (0–500 pg/ml) for 24 hours, then the phagocytosis of apoptotic cells by AMs was examined by flow cytometry. **(D, E)** AMs were stimulated with TNF-α (200 pg/mL) for 24 hours, then the surface expression levels of ADAM17 and CD36 were analyzed by flow cytometry. **(F)** AMs were stimulated with TNF-α (200 pg/mL) for 24 hours, and ELISA was used to measure the levels of sCD36 in the cell supernatant. **(G, H)** OVA-induced mice were subjected to intranasal treatment with TNF-α antibody, then ADAM17 and CD36 surface expression levels on AMs were analyzed by flow cytometry. **(I)** OVA-induced mice were subjected to intranasal treatment with TNF-α antibody, then the phagocytosis of apoptotic cells by AMs was assessed by flow cytometry. **(J)** OVA-induced mice were subjected to intranasal treatment with TNF-α antibody, and ELISA was used to measure the levels of sCD36 in BALF. **(L)** Normal control AMs were transfected with ADAM17-siRNA for 48 hours, then stimulated with 200 pg/ml TNF-α for 24 hours, and ELISA was used to measure the levels of sCD36 in the cell supernatant. **(K, M)** Normal control AMs were transfected with ADAM17-siRNA for 48 hours, then stimulated with 200 pg/ml TNF-α for 24 hours. Surface CD36 expression levels **(K)** and the phagocytosis of apoptotic cells by AMs **(M)** were analyzed by flow cytometry. For **(B, I, M)** gating strategy is described in Methods. **(D, E, G, H, K)** Gating strategy: The gating strategy for CD36 and ADAM17 was the same as that described for **Figures 2A–D**. In **(B, D, E, G–I, K, M)** the left panels display representative flow cytometry plots, and the right panels show statistical graphs. In **(H)** the upper panel displays a representative flow cytometry plot, and the lower panel shows a statistical graph. Control: normal control group, OVA: OVA-induced group, OVA+TNF-α Ab: OVA-induced group treated with TNF-α antibody, OVA+Iso Ab: OVA-induced group treated with isotype antibody, TNF-α: TNF-α stimulation alone group, TNF-α+si-ADAM17: TNF-α stimulation combined with ADAM17-siRNA transfection group, TNF-α+si-NC: TNF-α stimulation combined with control siRNA (scrambled sequence) transfection group. n=3, *P < 0.05, **P < 0.01, ***P < 0.001,****P < 0.0001.

Asthma, a chronic inflammatory airway disease, is associated with the release of multiple inflammatory mediators during its progression. To investigate the role of TNF-α, a known regulator of macrophage phagocytic function (15)in AMs-mediated phagocytosis, we first analyzed TNF-α levels in the BALF of different treatment groups of mice. Flow cytometry analysis revealed a significant increase in TNF-α levels in OVA-induced mice compared to wild-type controls (**Figure 4A**). Next, the impact of TNF-α on phagocytosis was determined by treating AMs from wild-type mice with TNF-α at concentrations ranging from 10 to 500 pg/ml for 24 hours. We found that TNF-α treatment markedly suppressed the phagocytic capacity of AMs, with the most significant inhibition observed at 200 pg/mL (**Figures 4B, C**). Flow cytometry analysis revealed that treatment with 200 pg/ml TNF-α significantly upregulated ADAM17 surface expression levels (**Figure 4D**), while downregulating CD36 expression levels (**Figure 4E**) and the level of sCD36 in the supernatant of AMs was upregulated (**Figure 4F**) compared to untreated controls. The effects of TNF-α on AMs-mediated phagocytosis were next examined *in vivo* by isolating AMs from OVA-induced mice following TNF-α blockade and quantifying their ADAM17 and CD36 surface expression levels, as well as the level of sCD36 in BALF. Compared to the isotype-matched control antibody, TNF-α blockade significantly downregulated ADAM17 and upregulated CD36 expression levels in the OVA+TNF-α Ab group (**Figures 4G, H**), along with a reduction in the level of sCD36 in BALF (**Figure 4J**). The functional consequences of TNF-α blockade were next determined by quantifying the phagocytic capacity of AMs from OVA-induced mice. Flow cytometry analysis revealed that TNF-α blockade significantly enhanced the phagocytic capacity of AMs in the OVA+TNF-α Ab group compared to the isotype-matched control antibody (**Figure 4I**). Next, we sought to elucidate the mechanism by which TNF-α regulates AMs-mediated phagocytosis by knocking down ADAM17 expression in AMs *in vitro*. Then, we observed the effects of TNF-α on the expression of CD36 receptors on the surface of AMs, the content of sCD36 in the cell supernatant, and the phagocytic function of AMs.The results revealed that CD36 expression levels were significantly upregulated (**Figure 4K**), the content of sCD36 decreased (**Figure 4L**), and the phagocytic capacity was markedly enhanced (**Figure 4M**) in the TNF-α+ADAM17-siRNA group compared to the TNF-α+si-NC group. These results demonstrated that knockdown of ADAM17 in AMs attenuated the TNF-α-induced downregulation of CD36 expression and suppression of phagocytic capacity. Collectively, our findings indicated that TNF-α upregulated ADAM17 to reduce CD36 surface expression, thereby impairing phagocytosis by AMs.

## 4 Discussion

Our study showed that AMs from OVA-induced mice had significantly impaired phagocytic capacity. phagocytosis by AMs is mediated by the specific recognition of apoptotic markers, such as PS, through Phagocytic receptors. CD36, a class B scavenger receptor, is essential for the direct recognition of PS (16). Consistent with this, CD36 deficiency has been shown to impair macrophage phagocytosis of apoptotic neutrophils (17), whereas CD36 upregulation has been shown to promote phagocytosis during wound healing (18). This study, focusing on the OVA-induced type 2-high asthma subtype, demonstrates for the first time a significant downregulation of CD36 surface expression on AMs in this model. Furthermore, CD36 blockade experiments establish a causal link between CD36 loss-of-function and impaired phagocytic capacity of AMs. Interestingly, indirect PS-binding receptors, such as MERTK, were not significantly changed in OVA-induced AMs, warranting further investigation into their functional roles.

The ADAM (a disintegrin and metalloproteinase) family, which includes both transmembrane and secreted proteins, plays important roles in regulating phagocyte phenotypes. The functions of individual ADAM members exhibit significant specificity depending on the pathological microenvironment (19). For example, ADAM12 promotes efferocytosis and polarizes macrophages toward an immunosuppressive M2 phenotype in a tumor microenvironment via an AXL-dependent mechanism involving Gas6 secretion (20). ADAM10 is activated upon LPS stimulation and enhances macrophage activation and proliferation, thereby contributing to the regulation of inflammatory responses (21). In contrast, ADAM9 suppresses efferocytosis and inhibits polarization toward an anti-inflammatory M2 phenotype in the context of sepsis-induced acute lung injury (22).

ADAM17 functions as a sheddase by cleaving cell surface receptors (23). Its enzymatic activity is regulated in disease states, wherein ADAM17 is frequently overexpressed, thereby contributing to immune suppression (24). In our study, we observed elevated expression of ADAM17 on the surface of AMs from OVA-induced mice. ADAM17 has a broad range of substrate receptors, including several key receptors that regulate phagocytic function, such as CD222, MERTK, TREM2, CD36, and TIM-1/4 (25–27). Among these, CD36 is an important pattern recognition receptor that mediates phagocytosis (28). We examined the expression of phagocytosis-related receptors on AMs and found that the expression of CD36 receptor on the surface of AMs from asthmatic mice was downregulated, which exhibited a negative correlation with the expression of ADAM17. Using siRNA knockdown of ADAM17, we also demonstrated that ADAM17 had a role in negatively regulating CD36 expression. Following knockdown of ADAM17 inhibition, we found that both CD36 surface expression levels and the phagocytic capacity of AMs were significantly restored, indicating that ADAM17 downregulated CD36 receptor expression through proteolytic cleavage. Consistent with our findings, Daniela et al. have previously demonstrated that ADAM17 inhibition enhances phagocytosis in macrophage subpopulations, including microglia (29). Collectively, CD36 is a critical mediator through which ADAM17 regulates the phagocytic function of AMs in the OVA-induced asthma model.

TNF-α, a prototypical member of the TNF protein superfamily (30), plays a critical role in various pathological conditions, including inflammation, infection, and asthma, with markedly elevated TNF-α levels observed in affected tissues. As a central mediator of airway inflammation in asthma, TNF-α levels have also been shown to be significantly increased in the BALF of OVA-induced mice, as demonstrated in this study. Our findings with respect to the role of TNF-α in macrophage phagocytic function were consistent with previous studies. Specifically, McPhillips et al. reported that TNF-α inhibits macrophage-mediated clearance of apoptotic cells through phospholipase A2 and oxygen-dependent pathways, thereby impairing the resolution of inflammation (31). Similarly, Feng et al. demonstrated that TNF-α downregulates Gas6 activation, impairing the phagocytosis of apoptotic neutrophils by peritoneal macrophages (32). Here, we showed that TNF-α inhibited the phagocytic capacity of AMs in a dose-dependent manner. Furthermore, we demonstrated that the phagocytic capacity of AMs was partially restored following TNF-α blockade *in vivo*. Collectively, these findings indicated that TNF-α-mediated inflammation contributed to the impaired phagocytic capacity of AMs in OVA-induced mice.

We found that TNF-α upregulated ADAM17 expression in AMs, while downregulating CD36 expression. Furthermore, we showed that TNF-α blockade in the airways of OVA-induced mice led to decreased ADAM17 expression on the surface of AMs, together with an increase in CD36 expression levels. *In vitro*, our ADAM17 knockdown experiments revealed that TNF-α downregulated CD36 expression via ADAM17, thereby modulating the phagocytic capacity of AMs. These results underscore the essential function of the TNF-α–ADAM17–CD36 axis in alveolar macrophage phagocytic activity within type 2−high asthma, thereby offering new insights into the immune regulatory mechanisms characteristic of this asthma subtype. Our findings showed that ADAM17, a key downstream effector molecule of TNF-α, impaired AMs phagocytic function by downregulating the expression of the phagocytic receptor CD36, thereby contributing to the pathogenesis of OVA-induced asthma model. These findings supported the development of therapeutic strategies targeting the TNF-α signaling pathway (e.g. TNF-α antagonists) or ADAM17 activity (e.g. specific inhibitors), potentially restoring CD36-dependent phagocytosis and offering new therapeutic avenues for asthma.

**Figure 5:**
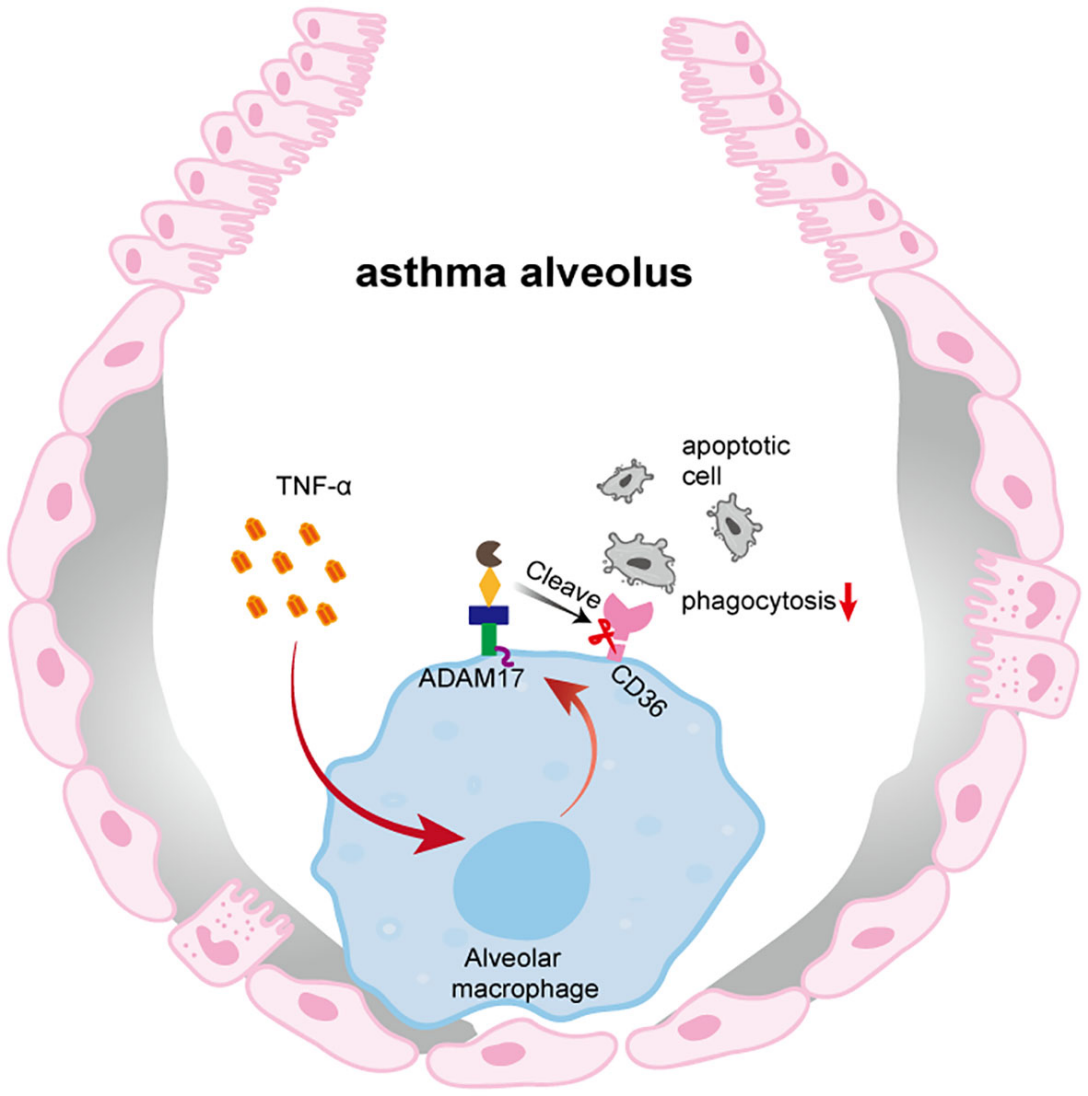
Impaired phagocytic capacity of AMs in OVA-induced mice. In the context of OVA-induced, TNF-α produced in the airways upregulates ADAM17 expression on the surface of AMs. ADAM17 cleaves the phagocytic receptor CD36 on AMs, thereby impairing their ability to phagocytose apoptotic cells.

In conclusion, our study demonstrated that TNF-α in the airways of OVA-induced mice upregulated the expression of ADAM17 and reduced the expression of CD36 on the surface of AMs, thereby impairing the phagocytosis of apoptotic cells by AMs (**Figure 5**). By focusing on the type 2-high asthma subtype, elucidating the distinct regulatory mechanism of CD36, and establishing a comprehensive *in vitro* and *in vivo* validation framework, this study provides systematic evidence for targeting phagocytosis-related mechanisms in asthma therapy. our findings will facilitate further exploration of the molecular mechanisms underlying the pathogenesis of asthma, as well as the identification of novel therapeutic targets for this disease.

## Data Availability

The raw data supporting the conclusions of this article will be made available by the authors, without undue reservation.
