# Supplementary material for: TNF-α-mediated downregulation of CD36 and phagocytic impairment of alveolar macrophages via upregulation of ADAM17 in asthma
Source: Front Immunol. 2025 Oct 16;16:1663513. doi: 10.3389/fimmu.2025.1663513 (PMC12571834; doi:10.3389/fimmu.2025.1663513)
Supplement: Supplementary file 1 [file DataSheet1.pdf]

Supplementary figures

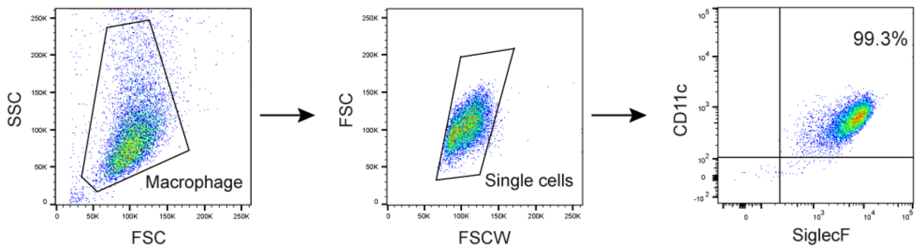

Figure S1. Gating strategy and purity confirmation of alveolar macrophages (AMs) via flow cytometry.

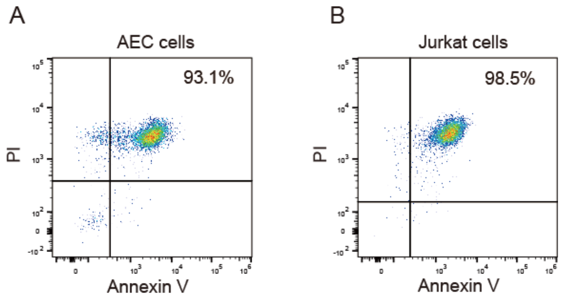

Figure S2: Analysis of camptothecin-induced apoptosis in AEC and Jurkat cells. (A)Flow cytometric analysis of apoptosis in AEC cells using Annexin V and propidium iodide (PI) staining. (B)Flow cytometric analysis of apoptosis in Jurkat cells using Annexin V and PI staining

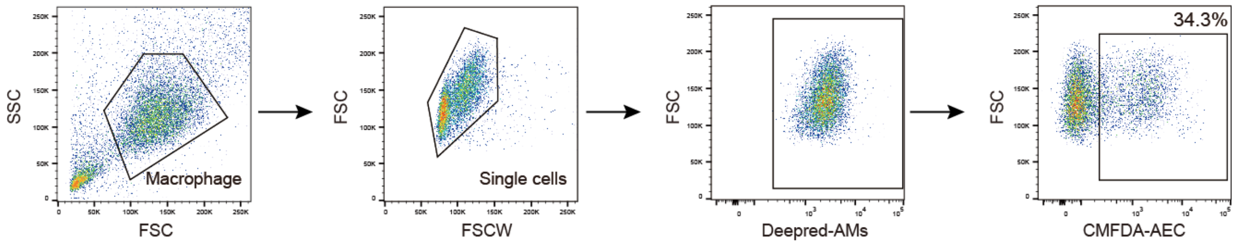

Figure S3. Representative flow cytometry gating strategy for identifying phagocytosing AMs (CMFDA^{+}/ Deep Red^{+}).
