# Supplementary material for: TNF-α-mediated downregulation of CD36 and phagocytic impairment of alveolar macrophages via upregulation of ADAM17 in asthma
Source: Front Immunol. 2025 Oct 16;16:1663513. doi: 10.3389/fimmu.2025.1663513 (PMC12571834; doi:10.3389/fimmu.2025.1663513)
Supplement: Supplementary file 2 [file Table1.docx]

**Supplementary tables**

| **Table S1. Sequences of si-RNAs** | |  |
| --- | --- | --- |
| Target | Sequence (5'-3') | |
| si-NC | UUCUCCGAACGUGUCACGUTT | |
| si-ADAM17 | GGAACUCUUGGAUUAGCUUTT | |
|  |  | |

| **Table S2. Primers used in qRT-PCR analyses** | |  |
| --- | --- | --- |
| GAPDH forward primer | ACCCTTAAGAGGGATGCTGC | |
| GAPDH reverse primer | CCCAATACGGCCAAATCCGT | |
| ADAM17 forward primer | CGTCGAGTGAAGAGACGAGC | |
| ADAM17 reverse primer | GTGCCTGAAATGTAGCCTAAGT | |
